# Non-genomic mechanisms of progesterone action in the brain

**DOI:** 10.3389/fnins.2013.00159

**Published:** 2013-09-19

**Authors:** Meharvan Singh, Chang Su, Selena Ng

**Affiliations:** Department of Pharmacology and Neuroscience, Center FOR HER, Institute for Aging and Alzheimer's Disease Research, University of North Texas Health Science Center at Fort WorthFort Worth, TX, USA

**Keywords:** progesterone, non-genomic, progesterone receptor, signaling, brain

## Abstract

Progesterone is a gonadal steroid hormone whose physiological effects extend well beyond the strict confines of reproductive function. In fact, progesterone can have important effects on a variety of tissues, including the bone, the heart and the brain. Mechanistically, progesterone has been thought to exert its effects through the progesterone receptor (PR), a member of the nuclear steroid hormone superfamily, and as such, acts through specific progesterone response elements (PRE) within the promoter region of target genes to regulate transcription of such genes. This has been often described as the “genomic” mechanism of progesterone action. However, just as progesterone has a diverse range of tissue targets, the mechanisms through which progesterone elicits its effects are equally diverse. For example, progesterone can activate alternative receptors, such as membrane-associated PRs (distinct from the classical PR), to elicit the activation of several signaling pathways that in turn, can influence cell function. Here, we review various non-nuclear (i.e., non-genomic) signaling mechanisms that progesterone can recruit to elicit its effects, focusing our discussion primarily on those signaling mechanisms by which progesterone influences cell viability in the brain.

## The biology of progesterone

Progesterone (Pregn-4-ene-3, 20-dione, P4), the natural progestin, is a major gonadal hormone that is synthesized primarily by the ovary in the female, and the testes and adrenal cortex in the male. While progesterone levels are generally higher in the female, it is worth noting that levels of progesterone during the female follicular phase of the menstrual cycle are similar to those seen in males (Strauss and Barbieri, 2004), and thus, may have important functions in males. Although the paradigmatic role for progesterone is on reproduction function, it has also been shown to exert significant extra-reproductive actions via multiple non-genomic signaling pathways. These different functions include immunomodulation (Hughes et al., 2013), inhibition of cholesterol biosynthesis (Metherall et al., 1996), and neuroprotection (Jodhka et al., 2009).

The “classical” mechanism by which progesterone elicits its effects is via the progesterone receptor (PR), which, like the estrogen receptor (ER), has classically been described as a nuclear transcription factor, acting through specific progesterone response elements (PRE) within the promoter region of target genes to regulate transcription. Two major isoforms of the classical PR exist, PR-B, and its N-terminally truncated form, PR-A [for review, see (Conneely and Lydon, 2000)]. The latter has been shown to exert negative control of not only PR-B-mediated transcription, but that mediated by the ER and glucocorticoid receptor as well (Vegeto et al., 1993). This negative regulation of ER function by a PR may underlie, at least in part, the mechanism by which progestins functionally antagonize the effects of estrogen. For example, progesterone can inhibit estrogen's ability to increase serum levels of 1, 25, dihydroxy vitamin D (Bikle et al., 1992), whose consequence may be to antagonize estrogen's beneficial effects on the bone. Relevant to post-menopausal hormone therapy, the functional antagonism exerted by progestins on estrogen's actions also underlie the rationale for combined estrogen and progestin therapy in women with an intact uterus, as the addition of a progestin reduces the risk of uterine cancer associated with un-opposed estrogen therapy (Hirvonen, 1996). However, the relationship between progesterone and ERs may not always be antagonistic. For example, Migliaccio et al. demonstrated not only a physical interaction of the PR with the ER, but that this association was necessary for progesterone to elicit the activation of a signal transduction pathway, the mitogen activated protein kinase (MAPK) pathway, in mammary tumor cells (Migliaccio et al., 1998).

While the classical mechanism by which progesterone elicits its actions is through the regulation of gene expression, progesterone has also been shown to elicit its effects via non-genomic mechanisms such as through the activation of signal transduction pathways, which in turn may be mediated by distinct PRs, including the more recently described membrane-associated PRs. Here, we review various non-nuclear signaling mechanisms by which progesterone can elicit its effects, focusing our discussion primarily on non-nuclear mechanisms by which progesterone influences cell viability in the brain.

## Diversity of signaling pathways that mediate the effects of progesterone

The classical PR-mediated cellular/physiological effects of progesterone are generally not rapidly elicited, given the time required to induce the transcription of genes and then, translate those genes into protein products. In contrast, it is now clear that progesterone can elicit rapid, non-genomic actions in various tissues including the brain through alternative mechanisms. These “non-classical” effects of progesterone can be initiated rapidly at the cell surface to activate intracellular signaling pathways, through modulation of putative cell surface receptors, ion channels, and cytoplasmic second messenger cascades. It is worth noting, however, that though these effects of progesterone are termed “non-genomic,” the rapid activation of cytoplasmic kinase signaling can result in both transcription-independent and transcription-dependent effects.

Among those rapid non-nuclear signaling pathways known to be activated by progesterone include the extracellular signal-related kinase (ERK) pathways (Migliaccio et al., 1998; Singh, 2001; Nilsen and Brinton, 2002; Boonyaratanakornkit et al., 2008; Su et al., 2012), cAMP/protein kinase A (PKA) signaling (Collado et al., 1985; Petralia and Frye, 2006), PKG signaling (Peluso, 2003), Ca^2+^ influx/PKC activation (Swiatek-De Lange et al., 2007), phosphatidylinositol 3-kinases (PI3 K)/Akt pathway (Singh, 2001; Zheng et al., 2012) and other signal transduction cascades. In addition, progesterone (or its metabolites) can act directly and rapidly on such neurotransmitter receptors such as the GABA-A receptor (Ishihara et al., 2013) and Sigma-1/2 receptors (Cai et al., 2008; Xu et al., 2011) to regulate cellular function.

With respect to calcium signaling, several reports suggest a functional link between progesterone and intracellular Ca^2+^ levels [(Ca^2+^)_*i*_]. For example, progesterone elicits increases in intracellular Ca^2+^ [(Ca^2+^)_*i*_] levels in *Xenopus* oocytes resulting in oocyte maturation (Wasserman et al., 1980). However, the cellular mediators involved in the progesterone-mediated changes in [Ca^2+^]_*i*_ remain to be determined. What is known is that intracellular Ca^2+^ channels (ICCs), such as IP3Rs, have been shown to be major components of the cytosolic Ca^2+^ regulation machinery (Berridge et al., 2000, 2003). Furthermore, several steroid hormones activate other signaling molecules that might in turn lead to activation of ICCs. In fact, both estradiol and progesterone elicit the phosphorylation of Akt in cerebral cortical cultures (Singh, 2001), a signaling protein that has been implicated in the phosphorylation of IP_3_Rs in Chinese hamster ovary T-cells (Khan et al., 2006). Progesterone, through an Akt-dependent pathway, can activate IP_3_R type 2, leading to enhancing channel activity of IP_3_R type 2 (Koulen et al., 2008; Hwang et al., 2009).

The consequences of activation of these signaling pathways are numerous and include influences on neurotrophin release (Su et al., 2012), neural progenitor proliferation (Liu et al., 2009), regulation of intracellular Ca^2+^ levels (Cai et al., 2008), and regulation of cell viability (Nilsen and Brinton, 2002, 2003; Kaur et al., 2007; Ishihara et al., 2013), all of which can contribute to the overall health and function of the brain.

## Receptors mediators of progesterone-induced signaling

**Figure 1:**
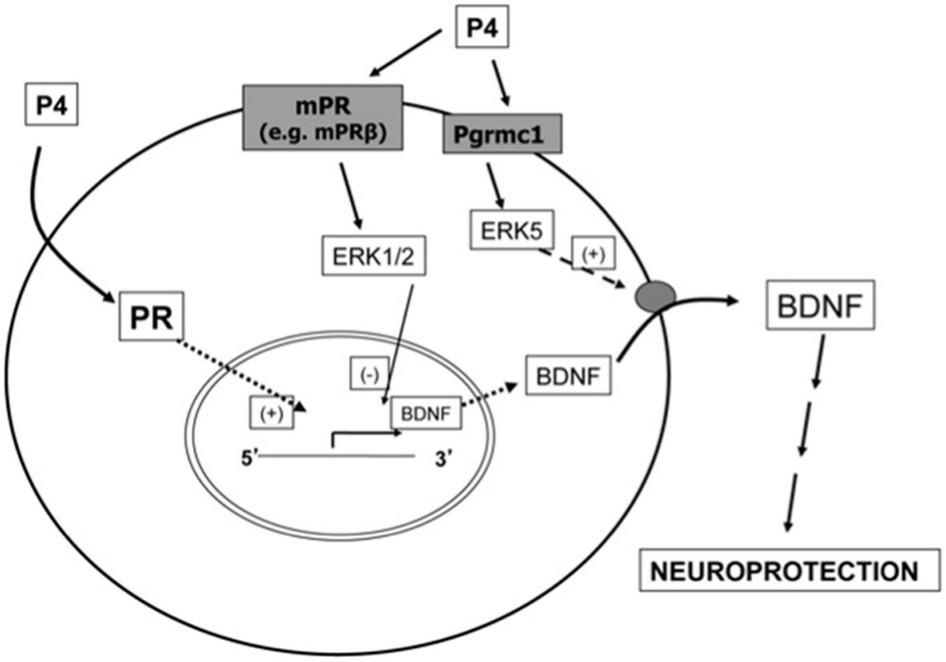
**Mechanism of progesterone action in the brain**. This figure provides a conceptual overview of how progesterone can elicit both genomic and non-genomic effects that impact its protective effects on the brain, and exemplifies how activation of complementary signaling cascades may be required for progesterone to fully elicit its effects. Using BDNF and two members of the ERK/MAPK family as paradigmatic examples of key mediators of progesterone-induced neuroprotection, this diagram underscores the fact that the protective effects of progesterone cannot be elicited by one signaling pathway alone, but rather, require complementary activation of multiple signaling pathways. In this example, the induction of BDNF synthesis requires the PR operating through its classical, genomic mechanism of action. However, such synthesis may not be meaningful unless the cellular content of BDNF can be secreted, leading in turn to the activation of its cognate receptors (ex. TrkB and/or p75) which can then elicit additional signaling pathways associated with cellular protection. This release of BDNF is mediated by a distinct receptor, Pgrmc1. In addition, other membrane-associated progesterone receptors, such as the mPR family of receptors, may elicit the activation of related signaling pathways that help finely regulate the process. In the example illustrated, activation of mPR may lead to activation of ERK1/2, which in turn, has been shown to exert an inhibitory influence on BDNF gene expression.

From a receptor pharmacology standpoint, the mechanism of progesterone action implicates the classical PR (e.g., PR-B or its N-terminally truncated variant, PR-A). Indeed, there are neuroprotective mechanisms of progesterone that require the classical PR. For example, our laboratory has determined that the ability of progesterone to increase the expression (mRNA and protein levels) of brain-derived neurotrophic factor (BDNF), a key mediator of progesterone's protective effects, requires the classical PR (Figure 1) (Jodhka et al., 2009). Further, Cai and colleagues (2008) have implicated the classical/intracellular PR in the protective effects of progesterone against an experimental model (middle cerebral artery occlusion) of stroke. However, evidence also exists for alternative mechanisms of action, including that which involves integral membrane PRs. For example, the effect of progesterone has been reported in the brain of PR knock-out mice (Krebs et al., 2000), suggesting PRs other than the classical PR may mediate the effect of progesterone in the CNS. In fact, several lines of evidence now support the role of cell membrane-associated PRs in mediating the effects of progesterone on the brain (Balasubramanian et al., 2008; Liu and Arbogast, 2009; Tokmakov and Fukami, 2009; Intlekofer and Petersen, 2011). The notion that membrane PRs exist is not new, and in fact, was supported by Hans Selye's pioneer work in the 40′s that showed various steroid hormones, including progesterone, had very rapid anesthetic effects in contrast to the delayed “main” hormone actions (Selye, 1942). Four decades later, specific, displaceable binding sites for progesterone were identified in synaptosomal membrane preparations (Towle and Sze, 1983; Ke and Ramirez, 1990). Further, progesterone's is quite lipophilic, having a log*P* value, or octanol/water partition coefficient, of 4. This value reflects the relative solubility of a compound in an organic phase (i.e., octanol) vs. an aqueous phase (e.g., water). As such, a log*P* value of 4 indicates that for every molecule of progesterone that partitions into the aqueous phase, 10,000 molecules partition into the organic phase. This further supports the idea that progesterone interacts with a plasma membrane-associated receptor.

Two types of distinct cell surface-associated proteins unrelated to classical PRs have been identified so far: membrane PRs (mPRs) and the progesterone membrane receptor component (PGMRC). The mPRs (molecular mass of approximately 40 kDa) had thought to be comprised of three subtypes, mPR α, β, and γ, which belong to the seven-transmembrane domain adiponectin Q receptor (PAQR) family (Zhu et al., 2003a,b). Two new subtypes, mPRδ and mPRε, have also been characterized recently in human brain (Pang et al., 2013). The mPRs bind to progesterone with high affinity (*K*_*d*_ ~5 nM) (Zhu et al., 2003a), and mediate important physiological functions in male and female reproductive tracts, liver, neuroendocrine tissues, and the immune system as well as in breast and ovarian cancer (Sleiter et al., 2009; Pang and Thomas, 2011). Uniquely, recent experimental evidence supports mPRs as G-protein-coupled receptors, as supported by the observation that activation of the mPRα can result in recruitment/activation of pertussis-sensitive inhibitory proteins (G_*i*_) to down-regulate membrane-bound adenylyl cyclase activity in the sea trout and in humans (Thomas et al., 2007).

Despite the fact that the classical PR and mPRs have overlapping regional expression (e.g., both are expressed in the hippocampus, cortex, hypothalamus, and cerebellum) (Brinton et al., 2008; Meffre et al., 2013), their profile of ligand specificity is not identical. For example, mPRs bind to 17α-hydroxyprogesterone and 5-dihydroprogesterone with greater affinity than to the classical PRs (Grazzini et al., 1998; Smith et al., 2008). In terms of cellular distribution, under non-injured conditions, the mPRα isoform was expressed principally by neuronal cells and not by oligodendrocytes or astrocytes. However, following traumatic brain injury (TBI) mPRα expression was observed in oligodendrocytes, astrocytes, and reactive microglia. This increase in mPR expression was proposed to mediate the anti-inflammatory effects of progesterone under conditions of injury (Meffre et al., 2013). Thus, the complement of PRs expressed in the brain may be driven by the health of the tissue.

In comparison to the mPRs, the single-transmembrane protein Pgrmc1 (molecular mass 25–28 kDa) and the closely related Pgrmc2 are thought to be a part of a multi-protein complex that binds to progesterone and other steroids, as well as pharmaceutical compounds (Thomas, 2008). Pgrmc1 was originally discovered in porcine liver and vascular smooth muscles (Falkenstein et al., 1996; Meyer et al., 1998), and later cloned in other species, including humans. Pgrmc1 has also been termed 25-Dx in rat and Hpr6 in human [for review, see (Cahill, 2007)], a result of being identified in different biological systems from multiple species. Pgrmc1 has an N-terminal transmembrane domain and a putative cytoplasmic cytochrome *b*5 domain ligand-binding motif. The cytoplasmic domain has target sequences for binding by SH2- and SH3-domain containing proteins as well as tyrosine kinases, implicating a potential role for Pgrmc1 as an adaptor involved in protein interactions and intracellular signal transduction. The subcellular localization of Pgrmc1 has been open to argument, since it was reported to localize in endoplasmic reticulum (Nolte et al., 2000), Golgi apparatus (Sakamoto et al., 2004) and nuclei (Beausoleil et al., 2004). However, evidence supporting the cell surface localization of Pgrmc1 includes the reports by Peluso et al., (Peluso et al., 2005) and ours (Su et al., 2012), in which biotinylated Pgrmc1 was localized to the surface (i.e., plasma membrane) of non-permeabilized cells.

Both mPRs and Pgrmc are expressed at high levels in the brain, but their functions relevant to progesterone effect in the CNS have only just started to be revealed. For example, a recent report demonstrated that allopregnanolone and other neurosteroids bound to mPRδ and decreased starvation-induced apoptosis in in hippocampal neuronal cells at low nanomolar concentrations (Pang et al., 2013). In addition, mPRα, mPRβ and Pgrmc1 have been implicated in progesterone-repressed gonadotrophin-releasing hormone release from hypothalamic neurons (Sleiter et al., 2009; Bashour and Wray, 2012). Progesterone-increased neural progenitor proliferation may also be mediated by Pgrmc1 as this effect was blocked by siRNA against Pgrmc1/2 (Liu et al., 2009). Further, a recent study by Frye, et al., revealed that progesterone-facilitated lordosis (sexual behavior) was significantly reduced by antisense oligodeoxynucleotides (AS-ODNs) against mPRβ, or AS-ODNs against both mPRβ and mPRα, when administered into the ventral tegmental area (VTA) (Frye et al., 2013). This data supports the potential role of mPRs in progesterone-facilitated lordosis of rats. The cell signaling pathways and associated downstream effects for progesterone-induced non-genomic actions are summarized in Table 1.

**Table 1:** **Receptor pharmacology and signaling pathways associated with progesterone-induced non-genomic effects**.

**Receptor**	**Signaling pathway**	**Effect**	**Species/cell/tissue type [references]**
PR	G_βγ_/adenylyl cyclase	Oocyte maturation	Xenopus oocyte [Guzman et al., 2005; Evaul et al., 2007]
PR	Src/ERK1/2/PI3K/Akt	Activation of transcription factors (e.g., Elk1)	Breast cancer [Saitoh et al., 2005; Fu et al., 2008]
PR	Src/RhoA	Inhibition of proliferation	Smooth muscle cells [Hsu et al., 2011]
Pgrmc1	ERK5	BDNF release	Glia [Su et al., 2012]
Pgrmc1, mPRα, mPRβ	N.D.	GnRH release	Hypothalamic neurons [Sleiter et al., 2009]
Pgrmc1/2	ERK	Neuronal progenitor proliferation	Dentate gyrus [Liu et al., 2009]
mPRα, mPRβ	N.D.	Lordosis	Ventral tegmental area [Frye et al., 2013]
mPRα	G_*i*_/adenylyl cyclase	N.D.	Sea trout, humans [Thomas et al., 2007]
mPRβ	MAPK	Oocyte maturation	Xenopus oocyte [Josefsberg Ben-Yehoshua et al., 2007]
N.D.	↑ [Ca^2+^]_*i*_	Oocyte maturation	Xenopus oocyte [Wasserman et al., 1980]

BDNF, brain-derived neurotrophic factor; ERK, extracellular-signal regulated kinase; GnRH, gonadotropin releasing hormone; MAPK, mitogen-activated protein kinase; mPR, membrane progesterone receptor; N.D., not determined; PI3K, phosphatidylinositol-3 kinase; Pgrmc, progesterone membrane component; PR, classical progesterone receptor.

The functions of membrane-associated PRs in the CNS are not limited to neurons. In fact, work from our laboratory supports that progesterone triggers BDNF release via Pgrmc1 signaling specifically from glia (Su et al., 2012). Another report showed that mPRα expression was induced in oligodendrocytes, astrocytes and reactive microglia after TBI (Meffre et al., 2013), supporting a potential role in mediating the effects of progesterone in inflammation and water homeostasis in the injured brain.

The two receptors do not always work independently. For example, Thomas and colleagues reported that activation of mPRα and -β in human myometrium leads to transactivation of PR-B (Karteris et al., 2006), as the first evidence that cross talk between the classical PR signaling and membrane-associated PR signaling exists.

It is also worth noting that the classical PR can also mediate the effects of progesterone on signaling pathways through non-genomic/extranuclear mechanisms of progesterone. Human PR-B contains a polyproline motif in its amino-terminal domain that interacts with the SH3 domain of Src (Boonyaratanakornkit et al., 2001). Therefore, cytoplasmic PR can mediate progesterone-induced rapid activation of c-Src and downstream Ras/Raf/ERK1/2 signaling independent of its transcriptional activity. Activation of the MAPK pathway ultimately results in the phosphorylation/activation of transcription factors such as c-Fos, c-Jun and nuclear PRs to control gene transcription. For example, progesterone was shown to inhibit aortic smooth muscle cell proliferation via Src phosphorylation that in turn, results in RhoA inactivation. The involvement of the PR was supported by the fact that this effect was blocked by RU486, a PR antagonist (Hsu et al., 2011). PR also mediates progesterone's effects on breast cancer development and progression by activating the Src/ERK1/2 or PI3K/Akt pathways (Saitoh et al., 2005; Fu et al., 2008), which leads to activation of the transcription factor Elk-1 and consequent changes in gene expression (Boonyaratanakornkit et al., 2008).

In addition to the well-characterized Src pathway downstream of extranuclear PR, there is evidence supporting the activation of G-protein signaling by PR in frogs (*Xenopus laevis*). For example, the *Xenopus* PR isoform related to the mammalian PR-B localizes to the plasma membrane of oocytes, and that activation of the PR regulates *Xenopus* oocyte maturation via the Gβ γ activation of adenylyl cyclase (Guzman et al., 2005; Evaul et al., 2007). Interestingly, another study concluded that the *Xenopus* ortholog of mPRβ mediated progesterone-induced oocyte maturation via the MAPK signaling (Josefsberg Ben-Yehoshua et al., 2007). Whether there is a cross talk between the PR/Gβ γ pathway and the mPRβ/MAPK pathway in this system remains unclear.

## Regulation of brain function through metabolites of progesterone

Another mechanism by which progesterone can exert protective effects is through its metabolites, which in turn, can interact with membrane-associated receptors coupled to ion-channels, such as the GABA_A_ receptor system [see (Deutsch et al., 1992) for review]. Such metabolites include allopregnanolone (or 3α, 5α tetrahydroprogesterone), which bind to discrete sites within the hydrophobic domain of the GABA_A_ receptor complex, and result in the potentiation of GABA-induced chloride conductance. Indeed, allopregnanolone has been suggested to play a role in mediating the protective effects of progesterone (Djebaili et al., 2004; He et al., 2004a,b; Vitarbo et al., 2004; Ardeshiri et al., 2006; Sayeed et al., 2009). In addition to the effects of allopregnanolone on the GABA_A_ receptor, as outlined above, allopregnanolone may also elicit its protective effects through its actions on the mitochondria (Robertson et al., 2006). For example, allopregnanolone was reported to inhibit currents associated with the opening of the mitochondrial permeability transition pore (mtPTP) (Sayeed et al., 2009), and as such, may help reduce the potential apoptotic consequences of mtPTP opening (such as cytochrome c release) during insult or injury. Moreover, allopregnanolone has also been shown to exert significant effects on neurogenesis [see (Wang et al., 2008) and references cited therein for review]. Interestingly, it has been shown that allopregnanolone may also elicit its protective effects through the regulation of BDNF [see (Nin et al., 2011) and references cited therein], although the precise mechanism by which allopregnanolone elicits BDNF [i.e., what receptor(s) allopregnanolone works through] is still unclear.

In addition to the allosteric effects described above, progesterone itself may have non-allosteric influences on the GABA_A_ receptor. Progesterone may influence the GABA_A_ receptor via the activation of a signal transduction pathway, which in turn, influences GABA-gated currents through phosphorylation of discrete sites within certain subunits of the GABA_A_ receptor (Vasan et al., 2003; Bell-Horner et al., 2006). Since the regulation of the GABA_A_ receptor has been shown to modulate cell survival, particularly in models of excitotoxicity, the regulation of the GABA_A_ receptor by progesterone may be relevant to the protective effect of progesterone seen against kainate-induced seizure activity and subsequent cell death (Hoffman et al., 2003).

And yet another non-classical by which progesterone can elicit its effects is through its interaction with the sigma 1 (σ_1_) receptor (Selmin et al., 1996; Seth et al., 1998). Given the reported role of the sigma 1 receptor in neuroprotection [for review, see (Maurice et al., 2006)], this mechanism may also be relevant to progesterone's protective actions.

Collectively, reports from numerous laboratories support the critical involvement of “non-genomic” signaling in mediating progesterone's effects, including its cytoprotective effects. This highlights not only the complexity by which progesterone exerts its effects on target tissues, but also reveals insight into discrete mechanisms that may be modulated for the purpose of developing novel therapeutic strategies.

### Conflict of interest statement

The authors declare that the research was conducted in the absence of any commercial or financial relationships that could be construed as a potential conflict of interest.
